# Molecular characterization of full fusion protein (F) of Newcastle disease virus genotype VIId isolated from Egypt during 2012-2016

**DOI:** 10.14202/vetworld.2018.930-938

**Published:** 2018-07-15

**Authors:** Karim M. Selim, Abdullah Selim, Abdelsatar Arafa, Hussein A. Hussein, Ahmed A. Elsanousi

**Affiliations:** 1 National Laboratory for Veterinary Quality Control on Poultry Production, Animal Health Research Institute, P.O. Box 264-Dokki, Giza 12618, Egypt; 2 Department of Virology, Faculty of Veterinary Medicine, Cairo University, Giza, Egypt

**Keywords:** cleavage site, fusion gene, heptad repeat domains

## Abstract

**Aim:**

The aim of this work was to study the full F gene sequence of Newcastle disease virus (NDV) in regard to pathotyping and genotyping and to study the evolution of this NDV in Egypt.

**Materials and Methods:**

The present study was conducted using samples from seven suspected NDV flocks of vaccinated chickens during 2012-2016 from six governorates in Egypt. The NDV was successfully isolated from pathological specimens through inoculation in specific pathogen-free embryonated chicken eggs.

**Results:**

Pathogenicity of the NDV isolates has been estimated through intracerebral pathogenicity index and ranged from 1.66 to 1.73 which indicates the velogenic type of NDV isolates. Pathotyping and genotyping of these isolates were done through sequencing of full-length F gene. Results indicated that the seven NDV isolates showed characteristic cleavage site motif (112RRQKRF117) for the velogenic strains of NDV. Phylogenetic analysis of the F gene clustered these isolates within Group I of genotype VIId within Israeli strains NDV/IS/2015, NDV-Ch/SD883, and most of the Middle East strains. Six of seven sequenced isolates have six potential N-linked glycosylation sites. The neutralization epitope on the five antigenic sites of fusion is conserved in all Egyptian strains of this study except NDV-KFR-B7-2012 which has a substitution at D 170 N in epitope A4. In all our strains, 10 cysteine residues are recorded, except one loss of cysteine at residue 370 in both NDV-EG-35-2014 and NDV-GHB-328F-2016.

**Conclusion:**

All viruses in this study have 52 amino acid substitutions within fusion gene in compared with Lasota strain that reveals importance for its antigenic and structural function. The present work highlights the important need to sequence F gene of NDV genotype VIId to investigate the evolution of this NDV in Egypt.

## Introduction

Newcastle disease (ND) is a highly contagious disease caused by Newcastle disease virus (NDV) that led to severe economic losses in domestic poultry. NDV belongs to the genus Avulavirus of family Paramyxoviridae. It was classified into two classes, Class I and Class II [1-3]. The World Organization for Animal Health (OIE) grouped the NDV as a notifiable list B disease with trade restrictions on infected areas or countries [4]. The genome of NDV has a single-stranded, negative-sense RNA genome of 15,200 nucleotides that contain six genes, which encode seven proteins. The matrix (M gene), the fusion (F gene), the hemagglutinin-neuraminidase (HN gene) and the RNA-dependent RNA polymerase (L gene) proteins, the nucleoprotein (NP gene), the phosphoprotein (P gene), and the V protein resulting from mRNA editing of the P gene [5,6,7].

The most virulent strains exhibited the consensus sequence formed from ^112^R/K-R-Q-R/K-R*F^117^ at the cleavage site of the F0 precursor, in contrast to ^112^G/E-K/R-Q-G/E-R*L^117^ in avirulent viruses [8]. The NDV F protein has two heptad repeat (HR) motifs in the F1subunit; HR1 is adjacent to the fusion peptide, HR2 is adjacent to the transmembrane (TM) domain, and F2 subunit contains HR3 [9]. Based on the analysis of the nucleotide sequence of the F gene, Class II virus is typically found circulating within wild bird and poultry species and has been divided into 18 genotypes (I–XVIII), with genotypes V–VIII being the most common genotypes circulating in the world [10,11]. Since ND was first described in 1926, three worldwide panzootics have occurred. The first panzootic (1940-1960) was caused by viruses belonging to genotypes II-III, the second (1960-1973) caused by VI, and the third (1970-1980) by genotypes V and VI [12]. Most of these isolates belong to genotypes V (North America and Africa), VI and VII (worldwide), XI (Madagascar), XII (Asia, South America), XIII (Asia), XIV (Nigeria), and recently designated genotypes XVI (Dominican Republic), and XVII and XVIII (Africa) [13]. Virulence of ND may also be distinguished on the basis of the cleavage site sequence of their F protein. During replication, the fusion gene is translated into a precursor protein, F0, which is cleaved by host cell proteases into F1 and F2 subunits to produce infectious viral particles [14]. In 2010, sequence analysis of some selected positive ND Egyptian viruses revealed that the circulating viruses were closely related to genotypes II and VI [15].

In another work, isolation and characterization of NDV from outbreaks in Fayoum, Behira, and Giza Governorates in Egypt from 2011 to 2012 were done, and the results indicated the presence of velogenic isolates of genotype VII subgenotype d NDV and closely related to the Middle East isolates [16]. NDV genotype VII was isolated from H5N1-infected Broiler Flock in 2012, and this isolate was in close range to Chinese strains [17].

The aim of this work was to study the sequence F gene of Newcastle disease virus (NDV) in regard to pathotyping and genotyping and to study the evolution of this NDV in Egypt.

## Materials and Methods

### Ethical approval

All applicable international, national, and/or institutional guidelines for the care and use of animals were followed.

### Sample collection

Organs (brain, trachea, lungs, proventriculus, small intestine, and pancreas) and swabs (cloacal and tracheal) were collected between 2012 and 2016 from seven chicken vaccinated flocks demonstrating high mortality and morbidity including respiratory, nervous signs, and diarrhea, in broiler, and drop in egg production with different percentage of mortality in layer and breeder. These samples were labeled and transported immediately on ice to the reference laboratory for quality control on poultry production (RLQP).

### Detection of NDV by real-time reverse transcriptase polymerase chain reaction (rRT-PCR)

The viral genomic RNAs of the selected samples were extracted using QiAamp Viral RNA Mini kit (Qiagen GmbH, Hilden, Germany) according to the manufacturer’s instructions, rRT-PCR was carried out using a commercial kit Quantitect Probe rRT-PCR kit (Qiagen, Inc., Valencia CA). Primers used NDV-F (F+4829) 5’-GGT GAG TCT ATC CGG ARG ATA CAA G-3’, NDV-R (F-4939) 5’-TCA TTG GTT GCR GCA ATG CTC T-3’ and probe- (F+4894) 5’-FAM-AAG CGT TTC TGT CTC CTT CCT CCA-BHQ-3’ [18]. The real-time rRT-PCR was conducted in the Stratagene 3005P MXpro Real-Time PCR System (Stratagene, USA).

### Virus isolation and propagation

Virus isolation was performed at the RLQP. Suspensions of organs and/or swabs were first centrifuged in a bench-top centrifuge at 4000 rpm for 5 min. Antibiotics (penicillin [2000 units/mL], streptomycin [0.01 mL/1 mL], gentamicin [50 µg/mL], and mycostatin [1000 units/mL]) were added to the supernatants and incubated for 1-2 h at room temperature [19].

A volume of 0.2 mL of the supernatant was inoculated into the allantoic cavity of five 9-11-day-old, specific pathogen-free (SPF) embryonated chicken eggs from Kom-Oushem, Fayoum SPF farm) according to the procedure described in the OIE Manual [19]. Then, eggs were incubated at 37°C for 4-7 days and observed daily. Allantoic fluid was harvested and tested for the presence of a hemagglutinating virus by the hemagglutination (HA) test. Samples that tested positive with the HA test were tested for the presence of NDV using standard polyclonal anti-NDV serum (GD) and standard H5 and H9 in hemagglutination inhibition (HI) test. Aliquots of NDV-positive allantoic fluid were stored at −70°C for RT-PCR and sequencing analysis [19].

### Intracerebral pathogenicity index (ICPI)

The pathogenic evaluation of the isolate was carried out using standard assay methods in specific isolators found in Lab animal experiment unit at NLQP to determine the ICPI. Briefly, 1-day-old chicks were inoculated intracerebrally with 0.1 ml of a 1:10 dilution of infective allantoic fluid. Chicks were monitored during the 8^th^ day observation period and scored as normal (0), sick or paralyze (1), and dead (2). Total scores were determined, and the mean daily score was calculated to obtain the ICPI. The ICPI values 0.7 or greater are identified as virulent by the OIE [20].

### RT-PCR for F gene of NDV

RT-PCR amplification was performed using Qiagen One-Step Enzyme Mix according to the manufacturer’s instructions, using primer sets for full F gene as shown in Table-S1. The electrophoresis of PCR products was done on ethidium bromide 1% stained agarose gel 1.5%, and the amplified products were visualized by gel documentation system - Image capture (Biometra, Germany).

**Table-S1:** Primers used for sequence of partial and complete fusion gene.

Primer name	Direction	Primers	Location	References
NDV-F330	Forward	AGG AAG GAG ACA AAA ACG TTT TAT AGG	330-357	NLQP
NDV-R700	Reverse	TCA GCT GAG TTA ATG CAG GGG AGG	700-676	NLQP
NDV-F640	Forward	CTA ACT GAA TTA ACT ACA GTA TTC GGG	640-667	NLQP
NDV-R1290	Reverse	GTC TAA TGA TAA GAC ATT GCA CGA ATG	1279-1290	NLQP
NDV-F1200	Forward	ATG TAC AGA CCC TCC TGG TAT CAT ATC	1200-1227	NLQP
NDV-R1740	Reverse	CTT AAG TCT TAT ACT TGA CAG GTT ATC	1713-1740	NLQP
NDV-M2	Forward	TGG AGC CAA ACC CGC ACC TGCGG		[41]
NDV-F2	Reverse	GGA GGA TGT TGG CAG CATT		[41]

NDV=Newcastle disease virus

### Genetic and phylogenetic analysis of F gene

Purified RT-PCR products were sequenced using Bigdye Terminator V3.1 cycle sequencing kit (Perkin-Elmer, Foster City, CA) and Applied Biosystems 3130 genetic analyzer machine (ABI, USA). Sequence similarities and relationships of the full fusion gene (7 samples) obtained in this study were compared with previously published NDV vaccine and reference strains available in the public database (BLAST, NCBI, USA). Amino acid phylogenetic tree was constructed for the sequenced samples in comparison to the previously published vaccine, reference, and Egyptian strains from the GenBank database using neighbor-joining method; bootstrapping at 1000 repeats using Mega 6 software [21]. Bioedit software (version 7.1) was used for alignment of the nucleotide and amino acid sequences [22]. Sequences generated in the frame of this study were submitted to the GenBank database with accession numbers as shown in Table-S2. N-linked glycosylation was detected using NetNGlyc 1.0 Server [23]. We created a three-dimensional structure for fusion monomer of avian NDV virus by SWISS-MODEL modeling online server [24] and visualized by PyMOL 1.1 program (DeLano Scientific LLC).

**Table-S2:** Database of the isolates in this study.

Code	Reception date	Governorate	Species	Production type	Accession number
NDV-KFR-B7-2012	January 19, 2012	Kafr el-sheikh	Chicken	Broiler	KX686723
NDV-ISM-460F-2013	June 5, 2013	Ismailia	Chicken	Broiler	KX686722
NDV-BEH-261F-2014	March 18, 2014	El Behara	Chicken	Breeder	KX686726
NDV-EG-35-2014	April 3, 2014	Sharkia	Chicken	Broiler	KX686724
NDV-GZ-339F-2015	March 19, 2015	Giza	Chicken	Breeder	KX686725
NDV-GZ-986F-2015	September 14, 2015	Giza	Chicken	Layer	KX686727
NDV-GHB-328F-2016	March 7, 2016	Gharbia	Chicken	Broiler	KX686728

NDV=Newcastle disease virus

## Results

### Detection of NDV and virus isolation

The seven samples were positive by rRT-PCR using specific primers and probe for NDV with threshold cycle between 16.4 and 24.38. Samples were isolated in SPF eggs, and the allantoic fluid was confirmed by HA and HI tests for differentiation among avian influenza and NDV. The seven selected isolates were positive for NDV by HI test and negative for avian influenza subtypes H9 and H5 as shown in Table-S3.

**Table-S3:** Results of virus isolation and purity using HA and HI tests.

Samples	Number of eggs	Daily candling post-inoculation					Cumulative dead/live	HA	HI ND	HI H5 and H9

		1	2	3	4	5				
NDV-KFR-B7-2012	5	1	4				5/5	+	+	-
NDV-ISM-460F-2013	5		5				5/5	+	+	-
NDV-BEH-261F-2014	5		5				5/5	+	+	-
NDV-EG-35-2014	5		5				5/5	+	+	-
NDV-GZ-339F-2015	5		5				5/5	+	+	-
NDV-GZ-986F-2015	5	1	4				5/5	+	+	-
NDV-GHB-328F-2016	5		5				5/5	+	+	-

NDV=Newcastle disease virus, HA=Hemagglutination, HI=Hemagglutination inhibition

### ICPI

The seven viruses of our study are characterized as virulent viruses according to ICPI as they ranged from 1.66 to 1.73 (Table-1). All seven viruses showed ICPI values >0.7.

**Table 1:** Pathogenicity of Egyptian NDV

Code	Pathotype	ICPI	Cleavage site
NDV-KFR-B7-2012	Velogenic	1.73	112RKQKR*F117
NDV-ISM-460F-2013	Velogenic	1.71	112RKQKR*F117
NDV-BEH-261F-2014	Velogenic	1.73	112RKQKR*F117
NDV-EG-35-2014	Velogenic	1.72	112RKQKR*F117
NDV-GZ-339F-2015	Velogenic	1.66	112RKQKR*F117
NDV-GZ-986F-2015	Velogenic	1.68	112RKQKR*F117
NDV-GHB-328F-2016	Velogenic	1.73	112RKQKR*F117

NDV=Newcastle disease virus, ICPI=Intracerebral pathogenicity index.

### Sequence and phylogenetic analysis of F gene

**Figure-1:**
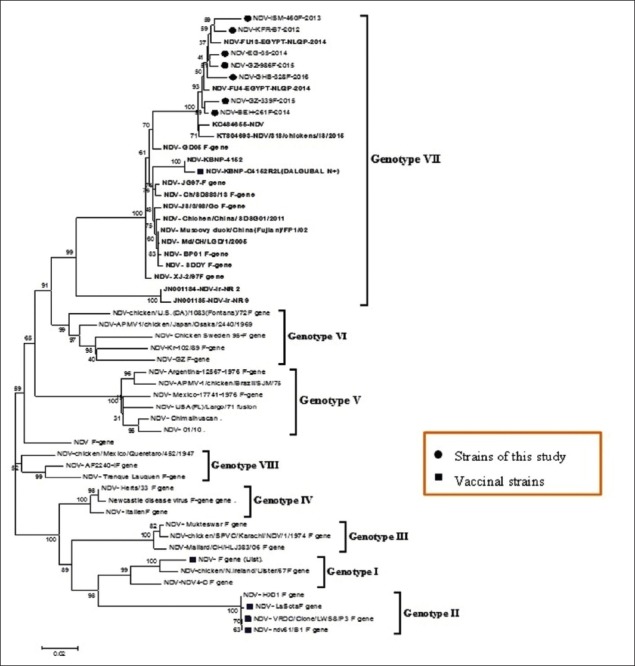
Phylogenetic tree for Newcastle disease virus (NDV) full F gene. Phylogenetic tree of F gene for (1700 pb; neighbor-joining method; bootstrapping 1000 repeats). Includes different international genotypes of NDV which are available in gene.

**Figure-2:**
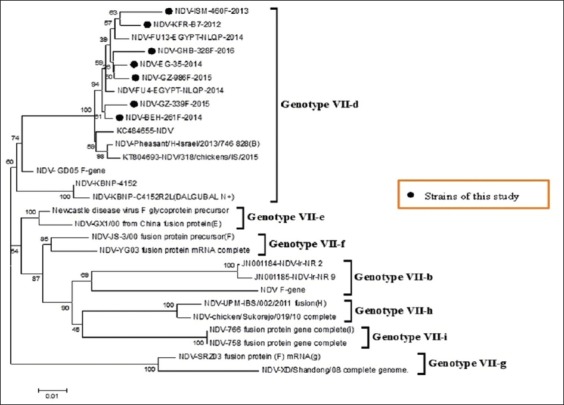
Subtyping of Egyptian selected isolates. Subtyping of Newcastle disease virus of seven Egyptian isolates with other reference strains result showed that selected isolates belonged to genotype VII-d.

Sequencing of seven confirmed NDV isolates for full fusion gene by RT-PCR assay revealed the expected and corrected size bands. The expected correct size of 400 bp for F1 fragment, 650 bp for F2 fragment, and 500 F3 fragment from the conventional RT-PCR products were detected and sequenced for full-length F gene to study the genetic and phylogenetic characterization of the Egyptian NDV viruses. All Egyptian isolates of the study were related to genotype VIId subtype, and they were very close to other Egyptian isolates with similarity percentage 98%, and with 94% similarity with other strains from China, Korea and Israel (Figures-1 and 2).

### Mutation analysis of fusion gene of NDV

**Figure-3:**
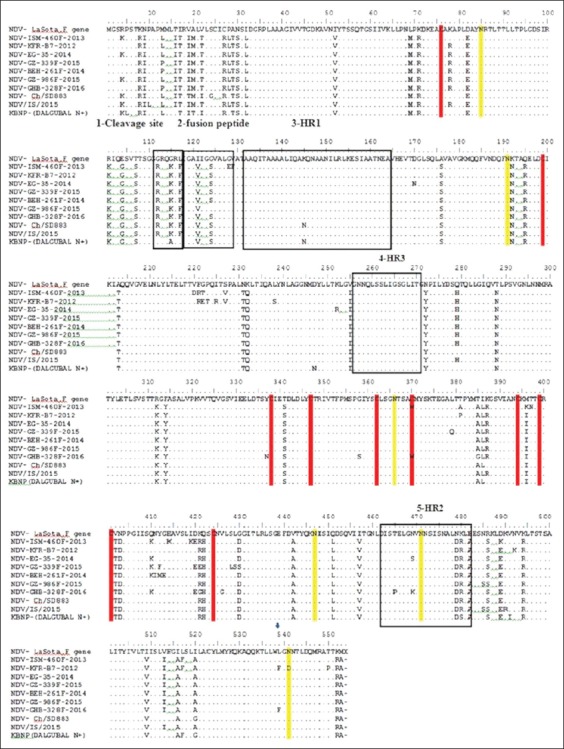
Mutation profile of fusion gene of Newcastle disease virus (NDV). This figure shows mutational substitution of the seven Egyptian isolates of the study in comparison with *Lasota*, *Israeli strains* NDV/IS/2015, and Chinese reference strain NDV-Ch/SD883 and genotype 7 vaccine (Korean vaccine dalguban N+). Red color column for cysteine residue, yellow color for N-glycosylation sites, and gray color for binding epitopes.

The seven isolates have 52 amino acid mutations in comparison to Lasota as shown in Figure-3, and these mutations recorded along fusion gene and include all different regions as the cleavage site, HR domains (HR1, HR2, and HR3), cytoplasmic domains, fusion peptide, and TM domains. According to the cleavage site sequence, all the Egyptian isolates in this study are velogenic as they have a typical characteristic motif 112RKQKR*F117 of the velogenic strain (Table-1).

The F protein of the seven viruses of NDV contains six potential acceptor sites for N-linked glycosylation at residues 85, 191, 366, 447, 471, and 541 (sites Ng1-Ng6, respectively). The sites at Ng2 and Ng5 are present in HR domains HR1 and HR2, respectively. In this study, some mutations were detected in HR1, HR2, HR3, and fusion peptide (Figure-3), and NDV-ISM-460F-2013 has a substitution from asparagine to aspartic acid (N541D) which led to loss in one glycosylation site (Ng6) in N-terminal portion of HR1.

There conserved 10 cysteine residues were recorded in the NDV strains of this study except two isolates (NDV-EG-35-2014 and NDV-GHB-328F-2016) that have a change in one cysteine residue 370 (C370W). The major epitopes involved in virus neutralization are conserved in most of the NDV strains of this study, except one isolate NDV-KFR-B7-2012 which has a substitution in epitope no 4 (A4) at amino acid residue 170 (D170N) as shown in Figure-3.

**Figure-4:**
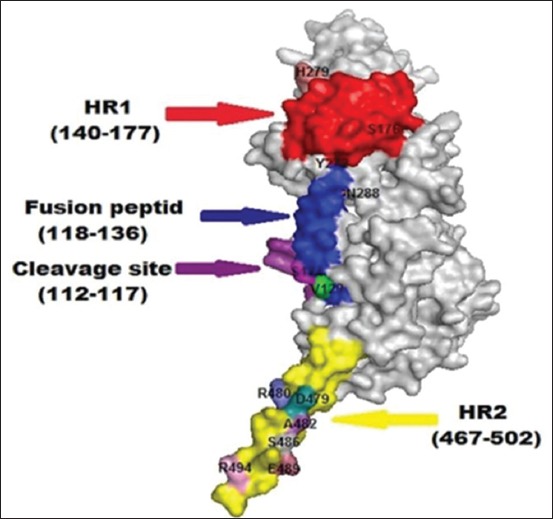
Three-dimensional structure for fusion monomer of avian Newcastle disease virus (NDV) (NDV-GZ-986F-2015 strain) was created by SWISS-Model modeling online server and visualized by PyMOL 1.1 program (DeLano Scientific LLC), Red color: HR1, Blue color: Fusion peptide. Pink color: Cleavage site, Yellow color: HR2.

The three-dimensional structure modeling for the full fusion protein (Figure-4), which represents the Egyptian stains of NDV in this study, showed the different active domains (HR1, HR2, cleavage site, and the fusion peptides region) and clarified the substitution residues in these domains in comparison to Lasota strain.

## Discussion

ND is a global disease of economic importance for domestic chickens. The NDV is capable of infecting a large number of avian species with varying degrees of clinical manifestations in different age groups [25]. Multiple NDV lineages are circulating worldwide, and most of them are considered as highly virulent. Outbreaks of NDV in vaccinated flocks have been increasingly reported around the world, suggesting suboptimal protection by vaccination. Hence, there are some vaccine breaks from genotypes I and II [26]. In Egypt, NDV outbreaks occurred in several vaccinated and non-vaccinated poultry farms in different governorates since 2011 causing severe respiratory and nervous signs and high mortalities that resulted in severe economic losses in 3-4-week-old broiler chickens [27,28].

In this study, the seven samples were positive for NDV inrRT-PCR. The seven positive samples were recorded from six governorates (Giza, Kafr el-Sheikh, Ismailia, Sharkia, Elbehara, and Gharbia) and from different time occurrences (January, March, April, June, and September) (Table-S2). Although the prevalence of ND in broiler and layer birds remains higher throughout the year, it reaches its plateau during seasonal stress (January–February, June–July, and September), and these results agree with the outbreak of ND which had killed 45 million chickens in Pakistan [29].

All samples were collected from vaccinated chicken farms including broilers, layers, and breeders. The viruses were reported from farms with different vaccination programs with high mortality percentage in these in broiler flocks and drop in egg production in layer and breeder flocks.

The seven samples were propagated in SPF ECE and then tested against the HA activity by HA assay, followed by HI assay to confirm the purity of these isolates from the other circulating hemagglutinating microorganisms as AI-H9N2 and H5N1 as shown in supplementary Table-S3.

Molecular pathotyping was conducted based on the results of the pathogenicity index (ICPI) and the amino acid sequences of the F protein proteolytic cleavage site (residues 112-117). The selected 7 viruses of this study reveal a velogenic type features with high pathogenicity index ranged from 1.66 to 1.73. The ICPI is a rapid and reliable method for NDV pathotyping in comparison to the mean death time and the intravenous pathogenicity index tests [30,31]. The ICPI values confirmed the high virulence of these viruses. The highest ICPI value was recorded in the previous work (1.97) where the virus placed in genotype VII, while other viruses from the same genotype gave ICPI values ranging from 1.7 to 1.87 [32] and the dibasic cleavage site motif of the very virulent viruses (^112^RKQKR*F^117^) as clarified in Table-1.

In this study, comparison of the fusion gene sequence of the Egyptian viruses with different NDV reference strains and available vaccines in Egyptian market revealed that all isolates have nucleotide similarity reached up to 98% with each other and other Egyptian isolates (NDV/FU4/Egypt,2014 and (NDV/FU13/Egypt 2014), Chinese (NDV- Ch/SD883), Israel strain (NDV/IS/2015, NDV/IS/2013 and NDV/IS/2011), and most Middle East strains except Iranian strain (NDV/Iran/NR24 and NDV/Iran/NR14) which belong to genotype VIIb. The Egyptian isolates have an identity with classic vaccine (heterologous vaccine) as Lasota, Clone, and Ulster strain about 80% but with genotype VII Korean vaccine which considers only homologous vaccine (C4152R2L) reached up to 94%.

As shown in Figure-3, more than 52 amino acid mutations were detected when comparing the selected seven Egyptian isolates with Lasota strain, and this will reveal on antibody production which will be a heterologous antibody, and this is at the level of F protein.

Phylogenetic analysis revealed that there was marked genetic distance between commercial ND vaccine and Egyptian isolates except genotype VII Korean vaccine (Dalguban N+) which located within the same group of the isolates of the study. Sequence analysis of fusion sequence showed that the selected seven Egyptian isolates are closely related to each other with the appearance of a minor new cluster within genotype VIId and this cluster as shown in Figures-1 and 2, and this may be due to evidence of mutation in fusion gene that was not found in Chinese or Israeli isolates (Figure-3).

Cattoli *et al*. [32] differentiated NDVs according to its cleavage site of fusion gene into different sublineages where sublineages (7a-, 7b- and 7d-group I) possessed four basic amino acids (RRQKR/F), while the viruses belonging to sublineages (7c- and 7d-group II) showed five basic amino acids (RRRKR/F). The seven Egyptian isolates in this study were related to genotype VIId Group I viruses from Israeli, Chinese, Korean, and other Middle East strains according to fusion protein cleavage site which contain basic amino acids (RRQKR/F).

The glycosylation process has a crucial role in viral glycoprotein folding and its function, which affects the viral infectivity, tropism, and antigenicity [33,34]. The most important N-glycosylation residues are located at positions 191 and 471 within the HR1 and HR2, respectively. Thus, the occurrence of changes at one of these 2 N-glycosylation residues might play an important role in the fusion promotion [35]. In this study, all N-linked glycosylation sites at residues 85, 191, 366, 447, 471, and 541 are conserved in all the selected Egyptian strains, except isolate NDV-ISM-460F-2013 that had a mutation in Ng6 (N541D) as shown in Figure-3. Previous studies suggested that the loss of this glycosylation residue solely had not increased the fusogenicity [35].

As the fusion protein is a surface protein on the NDV particles, there are common five antigenic epitopes on the fusion protein (A1-A5) [36,37]. In this study, the seven selected Egyptian viruses of genotype VIId have no changes in the whole antigenic epitope residues that related to this genotype, except NDV-KFR-B7-2012 which recorded a substitution at amino acid residue 170 (D170N) located in antigenic epitope A4 [38]. It was noticeable that the asparagine amino acid at residue 170 was recorded in genotype VI strains in Japan [39]. However, the A4 epitopes are located in N-terminus of HR1; structurally, this position makes these epitopes less exposed to the antibodies and consequently has low opportunities for induction of escape mutations [38].

Cysteine residues play a critical role in folding and provide structural stability to the protein through the formation of disulfide bonds [40]. There are 10 constant cysteine residues recorded in whole fusion of NDV in Egyptian strains of this study except the NDV-EG-35-2014 and NDV-GHB-328F-2016 that have a substitution in one cysteine residue 370 to tryptophan (C370W).

The paramyxovirus fusion proteins have a highly conserved leucine zipper motif immediately upstream from the TM domain of the F1 subunit. There was no change in leucine and isoleucine zipper has been shown as the five amino acids at the positions 767, 774, 778, 481, and 488 that give an indication about stability of confirmation of the fusion protein [35]. In this study, the selected Egyptian viruses of genotype VIId have no changes in the leucine zipper motif indicating the stability in its structure and configuration.

## Conclusion

The seven Egyptian NDV isolates in this work are related to genotype VIId Group I with alterations at different sites of the F gene interest as N-glycosylation sites, epitopes binding sites, and cysteine residues that may affect virus pathogenicity and may interfere with the classical vaccine protection. However, the fusion protein has several mutations that may affect its configuration, and this needs further study to link complete genome characterization with antigenic and pathogenic studies in relation to the available vaccine strains in the field.

## Authors’ Contributions

AS and KMS did the experiment. KM and AA did the molecular work. HAH and AAE supervised the technical work. KMS drafted and edited the manuscript. All authors read and approved the final manuscript.
